# A combined fibre/free-space-optical communication system for long-haul wireline/wireless transmission at millimetre-wave/sub-THz frequencies

**DOI:** 10.1038/s44172-023-00068-1

**Published:** 2023-05-03

**Authors:** Hai-Han Lu, Chung-Yi Li, Xu-Hong Huang, Cheng-Jun Lin, Ru-De Lin, Yu-Shen Lin, Yu-Sheng Tang, Wei-Cheng Fan

**Affiliations:** 1 Institute of Electro-Optical Engineering, National Taipei University of Technology, Taipei, 10608 Taiwan; 2 Department of Communication Engineering, National Taipei University, New Taipei City, 23741 Taiwan; 3 The School of Information Science and Engineering, Fujian University of Technology, Fujian, 350118 China

**Keywords:** Fibre optics and optical communications, Microwave photonics

## Abstract

The demands of the technologies of the future such as autonomous vehicles, Internet of Things and mixed reality require communications platforms equipped to handle huge quantities of data. Higher frequency communication bands are attractive but have limitations in terms of data loss particularly during wireless transmission. Free-space optical (FSO) communication, which transmits optical signals through free-space by modulating laser light, is one option for better wireless signal delivery. Here we report a platform combining multiple transmission media of 40-km single-mode fibre, with 1.2-km FSO communication, and short range (0.5-2 m) radio-frequency wireless. We demonstrate sufficiently low bit error rates and error vector magnitudes at sub-terahertz frequencies, satisfying the requirement of 5 G new radio communications applications.

## Abstract (web summary)

Communications systems delivering signals at millimetre-wave/sub-THz frequencies suffer capacity fading over both long distances fibre transmission and also over short distance wireless transfer. Hai-Han Lu and colleagues report a combined wired/wireless system with sufficiently low bit error rates and error vector magnitudes as to satisfy the needs of future 5 G communications technologies.

## Introduction

**Fig. 1:**
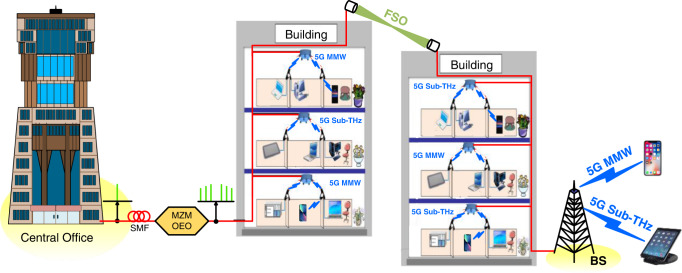
Transmission of MMW (MMW, millimeter-wave) and sub-THz (sub-THz, sub-terahertz) signals over combined fiber/FSO (FSO, free-space optical) communication systems. SMF single-mode fiber, MZM–OEO Mach–Zehnder modulator–optoelectronic oscillator, BS base station.

Looking ahead, we are moving towards innovative applications, such as emergency communications, mixed reality, self-driving cars, unmanned aerial vehicles, and the Internet of Things^1–5^. Nevertheless, these innovative applications require higher data rates, faster connections, and less latency. With recent development in millimeter-wave (MMW) and sub-terahertz (sub-THz) devices, it is no surprise that academics are pushing 5 G applications to higher frequencies, principally the MMW and sub-THz frequencies^6,7^. 5 G new radio (NR) MMW/sub-THz communication is gaining increasing attention due to its potential for enabling 5 G NR communication with high data rates^8–10^. However, 5 G NR communication faces an enormous challenge. For 5 G NR communication, high signal attenuation greatly limits its transmission distance. The 5 G NR communication limitations make it appropriate for dense/metropolitan districts. In rural/suburban districts, 5 G NR communication is not practical since it cannot transport signals over long distances. Free-space optical (FSO) communication uses laser light to transmit optical signals wirelessly through the air. It compensates for the high signal attenuation of 5 G NR MMW/sub-THz communication, thereby providing high-speed connections over long wireless distances^11–14^. Combining 5 G NR MMW/sub-THz communication, FSO communication at MMW/sub-THz frequencies will provide high data rates over long distances. Accordingly, the transmission of MMW and sub-THz signals over combined fiber/FSO communication systems for long-haul wireline/wireless transmission (see Fig. 1) shows the potential to provide high data rates. A combined fiber/FSO communication system at MMW/sub-THz frequencies can provide 5 G applications not only in dense/metropolitan areas but also in rural/suburban areas. It shows the prospect of 5 G applications aiming at dense/metropolitan and rural/suburban areas with high data rates.

A previous study presented the feasibility of constructing a fiber-FSO-5G NR MMW/sub-THz communication system utilizing a parallel/orthogonally polarized dual-carrier scheme^15^. The electrically generated 5 G NR sub-THz signal arises from the beating between dual carriers after photodiode (PD) detection. Establishing a fiber-FSO-5G NR MMW/sub-THz communication system with a coherent comb source was also proven to be feasible^16^. One optical carrier serves as an optical local oscillator located at the transmitting site. A local oscillator and another optical carrier are combined in a PD to generate an electrical MMW/sub-THz signal. However, due to the features of dual-/multi-carrier, dispersion-induced RF fading, and optical beating-induced interferences because of dual-/multi-carrier degrade link performance after 20 km single-mode fiber (SMF) transmission. In another study, academics enabled an MMW-over-fiber wireless link with a quantum dash dual-wavelength distributed feedback laser^17^. A 5 G NR fiber-wireless system using analog-over-fiber fronthaul to provide high-speed connectivity at MMW frequency. However, a combined fiber/FSO communication system at MMW/sub-THz frequencies has higher frequencies and can withstand higher data rates than a system at MMW frequency. Furthermore, there is room for further improvement of the 2–9 m MMW radio-frequency (RF) wireless, which can reach 1.2-km optical wireless link and 0.5–2 m MMW/sub-THz RF wireless.

In the following, we demonstrate a combined fiber/FSO communication system for long-haul wireline/wireless transmission at MMW/sub-THz frequencies with single optical carrier modulation to effectually decrease dispersion-induced RF fading and optical beating-induced interferences due to multi-carrier and coherent multi-carrier-optical-beating-to effectually decrease optical beating-induced nonlinear noises due to incoherent multi-carrier. Using 50, 100, and 150 GHz frequencies as scenarios, a combined fiber/FSO communication system at MMW/sub-THz frequencies is realized. Each MMW/sub-THz frequency transmits an 18.78-Gbps 16-quadrature amplitude modulation (QAM)-orthogonal frequency-division multiplexing (OFDM) signal. It is attractive because it avoids the need for a dispersion compensating device within the fiber transmission^18,19^ and shows potential for developing long-range 5 G NR communications with high data rates. Through 40 km SMF, 1.2 km optical wireless, and 2 m/1 m/0.5 m RF link, 5 G MMW/sub-THz 16-QAM–OFDM signals are transported with an impressive performance in terms of sufficiently low bit error rates (BERs) (<3.8 × 10^−3^ forward error correction (FEC) limit) and error vector magnitudes (EVMs) (<12.5% required limit). This combined fiber/FSO communication system for long-haul wireline/wireless transmission uses multiple transmission media to transport MMW and sub-THz signals with good performance. The successful demonstration of the fiber/FSO communication system is an important step toward the realization of 5 G MMW/sub-THz communications.

## Results and discussion

### To transmit the same data rate at different MMW/sub-THz frequencies

In this work, we transmit the same data rate at different MMW/sub-THz frequencies. If each MMW/sub-THz frequency carries its own data, then there will be crosstalk that arises from the adjacent wavelengths. The generation of crosstalk will reduce the transmission performance^20^. Moreover, multiple OFDM transmitters or complex filtered-OFDM modulation^21^ are needed to build such a combined fiber/FSO communication system at MMW/sub-THz frequencies. However, multiple OFDM transmitters will increase the cost of fiber/FSO communication systems, and filtered-OFDM modulation will increase the complexity of fiber/FSO communication systems at MMW/sub-THz frequencies. Although we transmit the same data at different MMW/sub-THz frequencies, however, each MMW/sub-THz frequency carries an 18.78-Gbps data rate, which is close to the 20 Gbps peak data rate required by 5 G. By transmitting three 5 G signals at MMW/sub-THz frequencies, a combined fiber/FSO communication system with three separate 18.78 Gbps high data is realized. It is a promising communication system that extends 5 G communications to three separate 18.78 Gbps high data for long-haul wireline-wireless transmission. One realistic scenario where the same data is transmitted at three wireless frequencies is in critical communication systems, such as emergency services or military communications. In these systems, reliability is essential, and transmitting the same data on multiple bands can increase the likelihood of successful transmission and reception.

### Configuration of Mach–Zehnder modulator (MZM) optoelectronic oscillator (OEO) and optical spectra

**Fig. 2:**
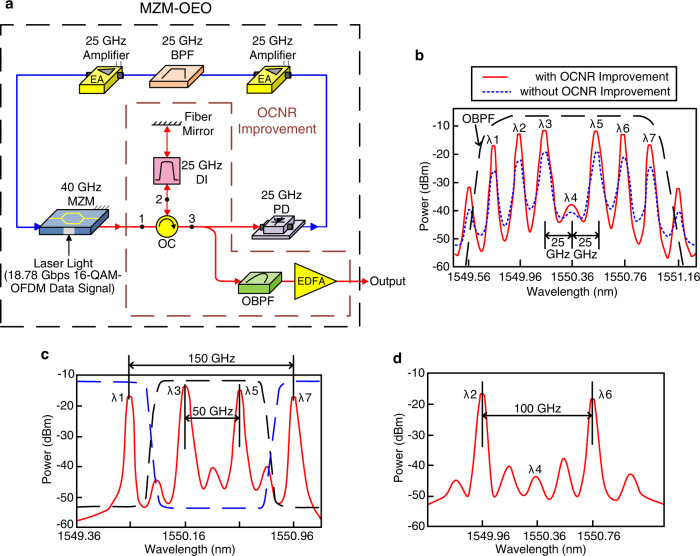
Configuration of MZM (MZM, Mach–Zehnder modulator)–OEO (OEO, optoelectronic oscillator) and optical spectra. **a** MZM–OEO (black dashed line) consists of a 40-GHz MZM, an OCNR (OCNR, optical carrier-to-noise ratio) improvement scheme (brown dashed line), a 25-GHz PD (PD, photodiode), and a 25-GHz BPF (BPF, band-pass filter) placed between two 25-GHz amplifiers. **b** MZM OEO’s output spectrum with (red line)/without (blue dashed line) OCNR improvement scheme. **c** Optical spectra of OIL (OIL, optical interleaver) output with odd optical carriers. By adopting an OBPF (OBPF, optical BPF) (black dashed line), a 50-GHz optical MMW (MMW, (MMW, millimeter-wave) signal is acquired. By adopting an OBSF (OBSF, optical band-stop filter) (blue dashed line), a 150-GHz optical sub-THz (sub-THz, sub-terahertz) signal is attained. **d** A channel spacing of 100 GHz is obtained, indicating that a 100-GHz optical sub-THz signal is achieved. 16-QAM-OFDM 16-quadrature amplitude modulation-orthogonal frequency-division multiplexing, DI delay interferometer, OC optical circulator, EDFA erbium-doped fiber amplifier.

As presented in Fig. 2a, the MZM OEO consists of a 40-GHz MZM biased at the null-bias point, an optical carrier-to-noise ratio (OCNR) improvement scheme, a 25-GHz PD, and a 25-GHz band-pass filter (BPF) placed between two 25-GHz amplifiers. Since MZM operates at the minimum transmission point, the light is operated in an optical carrier-suppressed format^22^. Part of the output is used as a light source with multiple optical carriers, whereas another part of the output is utilized for feedback through the optoelectronic feedback loop. The RF signal is firstly boosted by a 25-GHz amplifier, then filtered by a 25-GHz BPF and boosted by a 25-GHz amplifier, and finally sent to the MZM. MZM is driven by a 25 GHz RF signal to generate multi-carrier spaced 25 GHz apart. Note that the number of the generated optical carriers is directly proportional to the RF power level. As for the channel spacing, it is determined by the center frequency of the BPF. A 25-GHz BPF results in a 25-GHz channel spacing. These generated multiple optical carriers are sent to an OCNR improvement scheme to improve multiple optical carriers’ OCNRs. The OCNR improvement scheme includes an optical circulator, a delay interferometer, a fiber mirror, an optical BPF (OBPF), and an erbium-doped fiber amplifier (EDFA). The free spectral range of the delay interferometer is 25 GHz, the reflectivity of the fiber mirror is 98.5%, and the 3-dB bandwidth of OBPF is 1.4 nm. OBPF is employed to reject the outer optical carriers and noise, and EDFA is employed to boost multiple carriers filtered by the OBPF.

Figure 2b presents MZM OEO’s output spectrum with/without the OCNR improvement scheme. With the OCNR improvement scheme, the optical carrier’s OCNR is greatly improved by about 7 dB. We periodically set the generated multiple optical carriers according to the delay interferometer’s free spectral range to improve the OCNR. A delay interferometer acts as a power suppressor to suppress the power between optical carriers. We then utilize an EDFA to amplify multiple optical carriers filtered by an OBPF. When these filtered optical carriers pass through the EDFA, they are magnified so as to enhance the OCNRs. In addition, Fig. 2c, d exhibits the optical spectra of optical interleaver output with odd and even carriers, respectively. Clearly, a channel spacing of 50 GHz is obtained in Fig. 2c. By adopting a 0.6-nm OBPF, a 50-GHz optical MMW signal is acquired. By adopting a 1.1-nm optical band-stop filter, a 150-GHz optical sub-THz signal is attained. Furthermore, a channel spacing of 100 GHz is obtained in Fig. 2d, indicating that a 100-GHz optical sub-THz signal is achieved.

### Dispersion-induced RF fading and optical beating-induced interferences because of multi-carrier

For an external modulator with *α* chirp parameter, the photo-detected current *I*_*PD*_ can be obtained as^23^:

1 $${I}_{RF}\propto \sqrt{1+{\alpha }^{2}}\,\cos {\left[\frac{\pi LD{\lambda }^{2}}{c}{f}_{RF}^{2}+\arctan (\alpha )\right]}$$

where *L* is the fiber link, *D* is the dispersion parameter, *λ* is the wavelength, *c* is the light speed, and *f*_RF_ is the modulating signal frequency. With multi-optical carrier modulation, dispersion-induced RF fading reduces the photo-detected current and thereby degrades the transmission performance, resulting in worse BER and EVM. However, for single optical carrier modulation, there is almost no RF fading because dispersion is greatly reduced, leading to better BER and EVM.

By adopting multi-optical carrier modulation through 40-km SMF transport, the associated square law detection can be obtained as^24,25^:

2 $$|O{C}_{x1}	+ O{C}_{x2}+O{C}_{x3}+O{C}_{x5}+O{C}_{x6}+O{C}_{x7}|^{2}={|O{C}_{x1}|}^{2}\\ 	+ {|O{C}_{x2}|}^{2}+\cdots +{|O{C}_{x7}|}^{2}+2{{{{{\rm{Re}}}}}}\{O{C}_{x1}\cdot O{C}_{x2}\}\\ 	+ 2{{{{{\rm{Re}}}}}}\{O{C}_{x1}\cdot O{C}_{x3}\}+\cdots +2{{{{{\rm{Re}}}}}}\{O{C}_{x6}\cdot O{C}_{x7}\}$$

where *OC*_*x*1_, *OC*_*x*2_, *OC*_*x*3_, *OC*_*x*5_, *OC*_*x*6_, and *OC*_*x*7_ are optical carriers generated by MZM OEO (with optical carrier suppression format, *OC*_*x*4_ is suppressed). Obviously, interferences (*OC*_*x*1_∙*OC*_*x*2_, *OC*_*x*1_∙*OC*_*x3*_, …, *OC*_*x*6_∙*OC*_*x7*_) produced by optical beating due to multi-carrier reduce the link performance. Eliminating dispersion-induced RF fading and optical beating-induced interferences because of multi-carrier are vitally important for building a fiber-FSO-5G NR communication system with high link performance. To enhance the link performance of fiber-FSO-5G NR communication systems, we, therefore, adopt single optical carrier modulation through 40 km SMF transmission to efficiently reduce dispersion-induced RF fading and optical beating-induced interferences because of multi-carrier.

### Measured BERs as a function of optical power transmitted to PD/uni-traveling carrier (UTC)-PD and their associated constellations

**Fig. 3:**
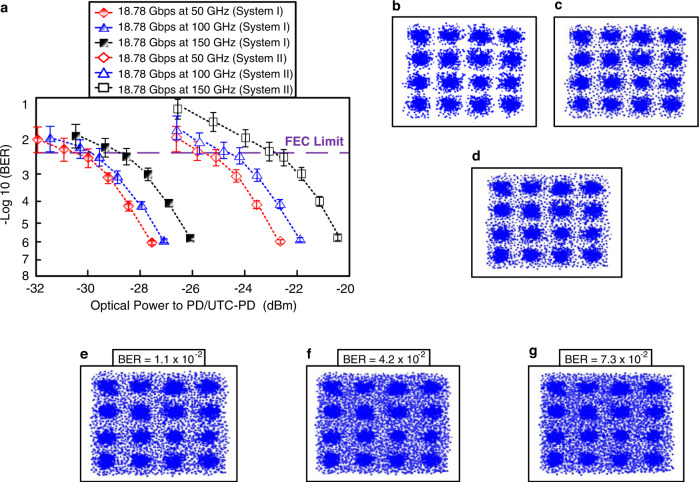
Measured BERs (BERs, bit error rates) and their associated constellations. **a** Measured BERs (BERs, bit error rates) as a function of optical power transmitted to PD (PD, photodiode)/UTC-PD (UTC-PD, uni-traveling carrier-photodiode) through 40 km SMF (SMF, single-mode fiber), 1.2 km optical wireless, and 2 m/1 m/0.5 m RF (RF, radio-frequency) wireless. The red rhombus/dashed line indicates 18.78 Gbps at 50 GHz (system I), the blue triangle/dashed line indicates 18.78 Gbps at 100 GHz (system I), the black square/dashed line indicates 18.78 Gbps at 150 GHz (the system I), red hollow rhombus/dashed line indicates 18.78 Gbps at 50 GHz (system II), blue hollow triangle/dashed line indicates 18.78 Gbps at 100 GHz (system II), and black hollow square/dashed line indicates 18.78 Gbps at 150 GHz (system II). The error bars represent the standard deviations of the measured data from three experimental trials. The associated constellations of 18.78 Gbps 16-QAM-OFDM (16-QAM-OFDM, 16-quadrature amplitude modulation-orthogonal frequency-division multiplexing) signal at **b** 50, **c** 100, and **d** 150 GHz carrier frequencies for the system I, when BER reaches 3.4 × 10^−3^. With multi-carrier modulation, degraded BERs of **e** 1.1 × 10^−2^, **f** 4.2 × 10^−2^, and **g** 7.3 × 10^−2^ with blurry constellations are obtained when the optical power transmitted to PD/UTC-PD is −27.2 dBm.

Figure 3a exhibits the measured BERs as a function of optical power transmitted to PD/UTC-PD through 40 km SMF, 1.2 km optical wireless, and 2 m/1 m/0.5 m RF link for systems I and II, respectively. For 18.78 Gbps 16-QAM-OFDM signal at 50, 100, and 150 GHz carrier frequencies, in system I, BER reaches 3.4 × 10^−3^ (<3.8 × 10^−3^ FEC limit) when the optical power transmitted to PD/UTC-PD is −29.8 (50 GHz carrier frequency), −29.2 (100 GHz carrier frequency), and −28.4 (150 GHz carrier frequency) dBm. In contrast, in system II, we obtain a BER of 3.4 × 10^−3^ when the optical power transmitted to PD/UTC-PD is −25.1 (50 GHz carrier frequency), −24.1 (100 GHz carrier frequency), and −22.2 (150 GHz carrier frequency) dBm. Large power penalties of 4.7, 5.1, and 6.2 dB exist between systems I and II. These large power penalties can be attributed to dispersion-induced RF fading because of 40 km SMF transport and optical beating-induced interferences because of multiple carriers. Fig. 3b–d shows the corresponding constellations of 18.78 Gbps 16-QAM-OFDM signal at 50, 100, and 150 GHz carrier frequencies for system I when BER reaches 3.4 × 10^−3^. Obviously, clear constellations (Fig. 3b), slightly clear constellations (Fig. 3c), and somewhat clear constellations (Fig. 3d) are achieved. When the carrier frequency increases, the phase noise increases as well. The phase noise of the 18.78 Gbps 16-QAM-OFDM signal at 50 GHz carrier frequency is lower than that of the 18.78 Gbps 16-QAM-OFDM signals at 100 and 150 GHz carrier frequencies. Compared with 18.78 Gbps 16-QAM-OFDM signal at 50 GHz carrier frequency, at the same BER, 18.78 Gbps 16-QAM-OFDM signals at 100 and 150 GHz carrier frequencies require higher optical power transmitted to PD/UTC-PD to compensate for the increase in phase noise. Such an increase in phase noise results in worse constellations. Impressive BERs (<3.8 × 10^−3^ FEC limit) and clear constellations support the transmission of MMW and sub-THz signals over combined fiber/FSO communication systems. Additionally, to verify the relation between the modulation format and the BERs, we investigate BERs and their correlated constellations of 18.78 Gbps 16-QAM-OFDM signal at 50, 100, and 150 GHz carrier frequencies for system II. When the optical power transmitted to PD/UTC-PD is −27.2 dBm, and the modulation format is multi-carrier modulation, degraded BERs of 1.1 × 10^−2^ (Fig. 3e), 4.2 × 10^−2^ (Fig. 3f), and 7.3 × 10^−2^ (Fig. 3g) with blurry constellations are obtained because dispersion-induced RF fading and optical beating-induced interferences are added to the SMF delivered optical carriers. It is to be observed that the center converges and the periphery diverges, indicating that the constellations’ noise distribution in Fig. 3e–g is non-Gaussian distribution, not Gaussian noise distribution. Since the communication system suffers from non-Gaussian noise, blurry constellations with unevenly distributed constellation points are acquired.

### Measured EVMs as a function of optical power transmitted to PD/UTC-PD and signal-to-noise ratio (SNR)

**Fig. 4:**
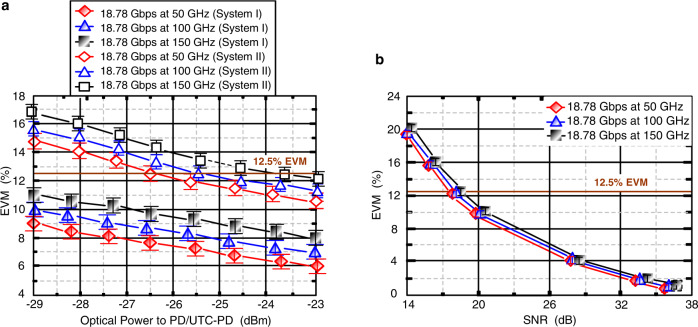
Measured EVMs (EVMs, error vector magnitudes). **a** Measured EVMs as a function of optical power transmitted to PD (PD, photodiode)/UTC-PD (UTC-PD, uni-traveling carrier-photodiode) over multiple transmission media of 40 km SMF (SMF, single-mode fiber), 1.2 km optical wireless, and 2 m/1 m/0.5 m RF (RF, radio-frequency) wireless. Red rhombus/line indicates 18.78 Gbps at 50 GHz (system I), blue triangle/line indicates 18.78 Gbps at 100 GHz (system I), black square/line indicates 18.78 Gbps at 150 GHz (system I), red hollow rhombus/line indicates 18.78 Gbps at 50 GHz (system II), blue hollow triangle/line indicates 18.78 Gbps at 100 GHz (system II), and black hollow square/line indicates 18.78 Gbps at 150 GHz (system II). **b** EVM as a function of SNR (SNR, signal-to-noise ratio) of the 5 G MMW (MMW, millimeter-wave)/sub-THz (sub-THz, sub-terahertz) 16-QAM-OFDM (16-QAM-OFDM, 16-quadrature amplitude modulation-orthogonal frequency-division multiplexing) signal for system I. Red rhombus/line indicates 18.78 Gbps at 50 GHz, blue triangle/line indicates 18.78 Gbps at 100 GHz, and black square/line indicates 18.78 Gbps at 150 GHz. The error bars represent the standard deviations of the measured data from three experimental trials.

Figure 4a presents the measured EVMs as a function of optical power transmitted to PD/UTC-PD through 40 km SMF, 1.2 km optical wireless, and 2 m/1 m/0.5 m RF link for systems I and II, respectively. In system I, the measured EVMs of 18.78 Gbps 16-QAM-OFDM signal at 50, 100, and 150 GHz carrier frequencies are less than the 12.5% requirement at all optical powers transmitted to PD/UTC-PD. In system II, nevertheless, the EVMs are less than the 12.5% requirement when the optical powers transmitted to PD/UTC-PD are higher than −26.5 (50 GHz carrier frequency), −25.4 (100 GHz carrier frequency), and −23.7 (150 GHz carrier frequency) dBm, respectively. The EVM degradation is mainly due to chromatic dispersion produced by the 40 km SMF transport and the interference produced by coherent multi-carrier beating. In addition, to closely correlate with SNR and EVM, the EVM as a function of SNR of the 5 G MMW/sub-THz 16-QAM-OFDM signal for system I is shown in Fig. 4b. Obviously, EVM is inversely proportional to SNR. EVM can be stated by EVM_*WN*_, EVM_*PhN*_, and EVM_*linearity*_ as^26^:

3 $${{{{{{\rm{EVM}}}}}}}^{2}={{{{{{{\rm{EVM}}}}}}}^{2}}_{{WN}}+{{{{{{{\rm{EVM}}}}}}}^{2}}_{{PhN}}+{{{{{{{\rm{EVM}}}}}}}^{2}}_{{linearity}}$$

where EVM_*WN*_, EVM_*PhN*_, and EVM_*linearity*_ are the EVMs related to white noise, phase noise, and nonlinear distortion, respectively. As noise and nonlinear distortion increase, EVM_*WN*_, EVM_*PhN*_, and EVM_*linearity*_ increase as well, resulting in an increase in EVM. SNR can be derived from EVM^27^:

4 $${{{{{\rm{SNR}}}}}}=-20{{\log }}({{{{{\rm{EVM}}}}}}/100 \% )$$

At 12.5% EVM, we observe SNRs of 17.8, 18.1, and 18.3 dB for an 18.78 Gbps 16-QAM-OFDM signal at 50, 100, and 150 GHz carrier frequencies, respectively. At an EVM threshold of 12.5%, 18.78 Gbps 16-QAM-OFDM signal at 50 GHz carrier frequency operates at a lower SNR than 18.78 Gbps 16-QAM-OFDM signal at 100 and 150 GHz carrier frequencies. At an SNR value of 18.3 dB, 18.78 Gbps 16-QAM-OFDM signal at 50 GHz carrier frequency operates at a lower EVM than 18.78 Gbps 16-QAM-OFDM signal at 100 and 150 GHz carrier frequencies. Since EVM_*WN*_ is directly related to peak-to-average power ratio (PAPR), an 18.78 Gbps 16-QAM-OFDM signal over 50 GHz lower carrier frequency has a lower PAPR, leading to a lower EVM_*WN*_. Moreover, since EVM_*PhN*_ is directly related to frequency, an 18.78-Gbps 16-QAM-OFDM signal over 50 GHz lower carrier frequency has less phase noise, leading to a lower EVM_*PhN*_. Lower EVM_*WN*_ and lower EVM_*PhN*_ result in an 18.78 Gbps 16-QAM-OFDM signal at 50 GHz carrier frequency with lower SNR at the same EVM and lower EVM at the same SNR.

In 5 G NR, subcarrier spacings of 15, 30, 60, 120, 240, 480, and 960 kHz are supported. Subcarrier spacing of 20 MHz is larger than that from 15 to 960 kHz. To closely correlate with subcarrier spacing and fiber-FSO-5G communication systems over MMW and sub-THz bands, subcarriers spaced by 240 kHz are used in the 16-QAM-OFDM signal. Over multiple transmission media of 40 km SMF, 1.2 km optical wireless, and 2 m/1 m/0.5 m RF link, for system I, BER reaches 3.8 × 10^−3^ (7% FEC limit) when the optical power transmitted to PD/UTC-PD is −26.4 (50 GHz carrier frequency), −25.7 (100 GHz carrier frequency), and −25.1 (150 GHz carrier frequency) dBm. Furthermore, the measured EVMs of the 18.78 Gbps 16-QAM-OFDM signal at 50 GHz carrier frequency are less than the 12.5% requirement at all optical powers transmitted to PD. And the measured EVMs of 18.78 Gbps 16-QAM-OFDM signal at 100 and 150 GHz carrier frequencies are less than the 12.5% requirement when the optical powers transmitted to PD/UTC-PD are higher than −27.2 (100 GHz carrier frequency) and −25.6 (150 GHz carrier frequency) dBm. Decreasing the subcarrier spacing decreases the strength of the system against intercarrier interference and brings on worse transmission performance in terms of higher BERs and EVMs^28,29^. However, low BERs and EVMs demonstrate the feasibility of combined fiber-FSO communication systems with single optical carrier modulation at subcarrier spacing compliant with 5 G NR requirements.

## Methods

### Combined fiber-FSO communication systems at MMW/sub-THz frequencies

**Fig. 5:**
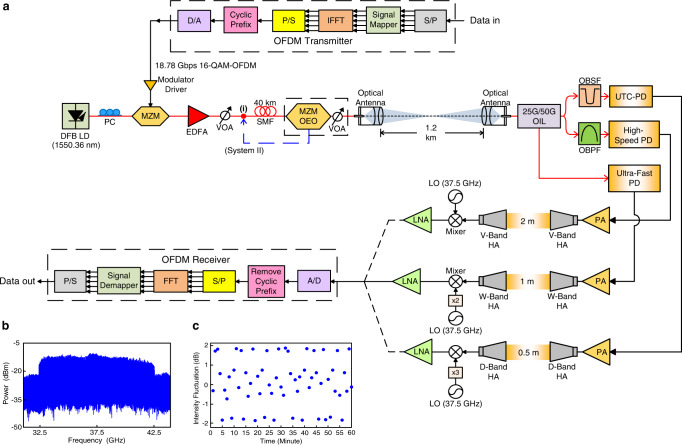
Combined fiber-FSO (FSO, free-space optical) communication systems. **a** Framework of combined fiber/FSO communication systems at MMW (MMW, millimeter-wave)/sub-THz (sub-THz, sub-terahertz) frequencies with single optical carrier modulation through 40 km SMF (SMF, single-mode fiber) transport and coherent multi-carrier beating after 40 km SMF transport (system I). **b** Electrical spectrum of 18.78 Gbps 16-QAM-OFDM (16-QAM-OFDM, 16-quadrature amplitude modulation-orthogonal frequency-division multiplexing) signal over 150 GHz carrier frequency through 40 km SMF, 1.2 km optical wireless, and 0.5-m RF (RF, radio-frequency) link (system I). **c** Intensity fluctuation due to laser light misalignment over 60 min. DFB LD distributed feedback laser diode, PC polarization controller, MZM–OEO Mach–Zehnder modulator-optoelectronic oscillator, EDFA erbium-doped fiber amplifier, VOA variable optical attenuator, OIL optical interleaver, OBPF optical band-pass filter, OBSF optical band-stop filter, PD photodiode, UTC-PD uni-traveling carrier-photodiode, PA power amplifier, HA horn antenna, LO local oscillator, LNA low noise amplifier, S/P serial-to-parallel, IFFT inverse fast Fourier transform, P/S parallel-to-serial, D/A digital-to-analog, A/D analog-to-digital.

Figure 5a illustrates the framework of combined fiber-FSO communication systems for long-haul wireline/wireless transmission at MMW/sub-THz frequencies with single optical carrier modulation through 40 km SMF transport and coherent multi-carrier beating after 40 km SMF transport (system I). Note that an actual experimental setup rather than a simulation is developed. A distributed feedback laser diode, with a 1550.36 nm center wavelength, provides an optical carrier to an MZM. The 18.78 Gbps 16-QAM-OFDM signal from the OFDM transmitter passes through the modulator driver and next drives the MZM. An EDFA enhances the optical carrier, and a variable optical attenuator adjusts the optical power supplied into the 40 km SMF. Via 40 km SMF transmission, an optical carrier modulated with 18.78 Gbps 16-QAM-OFDM signal is sent to an MZM OEO to generate multiple coherent carriers spaced 0.2 nm (25 GHz) apart. These produced optical carriers are next attenuated by a variable optical attenuator and transmitted through a 1.2-km optical wireless communication via a set of optical antennas^30^. The optical antenna at the receiving side concentrates the laser beam to maintain FSO communication with reliable free-space link^31,32^. By employing a 25 G/50 G optical interleaver, an OBPF, and an optical band-stop filter, an 18.78 Gbps optical signal at 50, 100, and 150 GHz carrier frequencies is generated. These optical signals are then transmitted to a high-speed PD with 50 GHz bandwidth, an ultra-fast PD with 100 GHz bandwidth, and a UTC-PD with 150 GHz bandwidth, respectively. Electrically generated 18.78 Gbps signal at 50, 100, and 150 GHz MMW/sub-THz frequencies originate from the beating between two carriers separated by 50, 100, and 150 GHz. These signals are amplified by three independent PAs with 50–75 GHz frequency, 75–110 GHz frequency, and 110-170 GHz frequency. Since the 16-QAM-OFDM signal is affected by PA’s nonlinearity and efficiency, PA is thereby operated with moderate linearity to reduce nonlinear distortions and moderate efficiency to mitigate power consumption. Next, the signals pass through a separate V-/W-/D-band horn antenna (HA) with frequency (GHz)/gain (dBi) of 50–75/25, 75–110/20, and 110–170/20. Through the 2 m/1 m/0.5 m RF link, there is a link budget of 29.9/34.2/32.1 dB between V-/W-/D-band HAs. The signals are down-converted by independent mixers with electrical local oscillators and frequency multipliers and enhanced by three independent low noise amplifiers with frequency (GHz)/P1dB (dBm)/noise figure (dB) of 0.3–20/20/3, 18–40/7/3.5, and 0.1–50/10/5. They are then subsequently analyzed by the OFDM analyzer for offline processing by MATLAB.

To have more associations with single/multiple optical carrier(s) modulation and link performance, we replace the location of MZM OEO to evaluate link performance. System II exhibits a fiber-FSO-5G NR MMW/sub-THz communication system with multi-carrier modulation. In system I, MZM OEO is placed after 40 km SMF transport. Multiple carriers are transmitted over a 1.2 km optical wireless link. In system II, MZM OEO is placed in front of 40 km SMF [inset (i) of Fig. 5a]. Multiple carriers are transported over 40 km SMF and 1.2 km optical wireless link.

Figure 5b presents the electrical spectrum of 18.78 Gbps 16-QAM-OFDM signal over 150 GHz carrier frequency through 40 km SMF, 1.2 km optical wireless, and 0.5 m RF link (system I). The electrical spectrum of 18.78 Gbps 16-QAM-OFDM signal over 37.5 GHz carrier frequency has slightly larger but acceptable amplitude fluctuations at ±3.8 dB, revealing the practicability of building fiber-FSO-5G NR communication systems. Furthermore, Fig. 5c shows the intensity fluctuation due to laser light misalignment over 60 minutes. A small intensity fluctuation of ±1.8 dB occurs over 60 minutes, revealing that this combined fiber/FSO communication system at MMW/sub-THz frequencies has a high link reliability over a long period of time.

### OFDM modulation/demodulation and the calculation of data rate

OFDM modulation utilizes a large number of subcarriers to carry data. The data is first converted from a serial format to a parallel format and then mapped to QAM symbols. These symbols are then input to an inverse fast Fourier transform (IFFT) operation, which yields a time-domain signal that is converted back to serial format using parallel-to-serial conversion. To eliminate inter-symbol interference caused by the channel, a cyclic prefix (CP) is inserted at the beginning of each OFDM symbol. The resulting signal is then converted to analog using a digital-to-analog converter for transmission. For OFDM demodulation, the received analog signal is first converted to digital using an analog-to-digital converter. The CP is then removed from the beginning of each OFDM symbol, and the resulting signal is converted to parallel format using serial-to-parallel conversion. An FFT block is then used to convert the time-domain signal to the frequency domain, and the resulting subcarriers are de-mapped to QAM symbols. The symbols are then converted back to serial format using parallel-to-serial conversion.

The signal goes through synchronization, estimation, and OFDM demodulation processes before calculating BER and EVM. The 16-QAM-OFDM signal has the parameters of 512 FFT size, 256 subcarriers (248 data subcarriers + 8 pilot subcarriers), 20 MHz subcarrier spacing, 16 CP samples, and 10 G samples per second (GSa/s). Out of these 256 subcarriers, 8 subcarriers are used for pilot signals. Pilot subcarriers do not carry modulated data but are used for channel estimation and equalization purposes. The remaining 248 subcarriers are used for carrying modulated data. Phase shift introduced by the channel is compensated for using the phase information from the pilot subcarriers. There are originally 512 samples in the data set. 16 CP samples are added to the data set. Thus, the total number of samples becomes 528. There are 248 carriers, and each carrier carries 4 bits of information. Therefore, the total number of bits transmitted per symbol is 248 × 4 = 992. The bit per sample rate can be calculated by dividing the total number of bits by the total number of samples: 992/528 = 1.878 bit/sample. The digital-to-analog conversion transmits the samples at a rate of 10 GSa/s. The data rate can be calculated by multiplying the bit per sample rate with the transmission rate: 1.878 × 10 = 18.78 Gbps. Therefore, the data rate achieved by transmitting the given set of samples is 18.78 Gbps.

### D-band HAs link budget calculation

For D-band HAs, the received power (*P*_*R*_) can be calculated by the Friis transmission equation^33,34^:

5 $${P}_{R}={P}_{T}+{G}_{T}+{G}_{R}-20{{\log }}\,(4\pi {df}/c)-{L}_{m}$$

where *P*_*T*_ is the transmitter power, *G*_*T*_ is the antenna gain of the transmitting side, *G*_*R*_ is the antenna gain of receiving side, *d* is the wireless distance, and *L*_*m*_ is the atmospheric loss. *P*_*T*_ is approximately 17 dBm, *G*_*T*_ and *G*_*R*_ are 20 dBi, and *d* is 0.5 m. The atmospheric loss *L*_*m*_ is approximately 2.1 dB for 0.5 m wireless distance at 150 GHz. The free-space path loss 20 log(4πdf/c) equals approximately 70 [20 log•(4*π*•0.5•(150 × 109)/(3 × 108))~70] dB. The received power *P*_*R*_ can be calculated as −15.1 dBm (17 + 20 + 20 − 82 − 2.1), and a link budget of 32.1(17−(−15.1)) dB can be obtained. The higher the link budget, the better the quality of the communication system. Compared to a lower link budget, a higher link budget of 32.1 dB results in improved performance in terms of low BERs and EVMs. Furthermore, it can be derived that the input of the low noise amplifier is much smaller than its input P1dB level (10 dBm), showing that the low noise amplifier is used in the linear region.

### Maximum optical wireless link calculation

The optical antenna, with a 75 mm diameter and 150 mm focal length, is a meniscus lens with a concave-convex lens. Since optical fiber’s numerical aperture is 0.15, the laser light’s diameter (*d*) is calculated as 45 mm (2 × (150 × 0.15)). On the transmitting side, the diameter of laser light is less than that of the optical antenna, leading to a feasible FSO link. Furthermore, the corresponding beam radius *r* (=2.3/((spatial frequency cutoff) × 2*π*)) and the laser light’s divergent angle ${\theta }$(=3.6 μm/150 mm) can be calculated to be 3.6 μm and 24 × 10^−6^, respectively. Through the *l*–m FSO link, the diameter of laser light (*d*_*L*_) should be less than that of the optical antenna (*d*_*L*_ < 75 mm) to yield a reliable FSO communication:

6 $${d}_{L}=\sqrt{{d}^{2}+{\left(2\theta l\right)}^{2}}=\sqrt{{45}^{2}+{\left(0.048l\right)}^{2}} < 75$$

*l* is derived as 1250 m, revealing that the maximum FSO link is 1250 m. The FSO link performed in such an operation is 1200 m (<1250 m) to satisfy the maximum optical wireless link demand.

### Supplementary information

Description of Additional Supplementary Files

Supplementary Data 1

Supplementary Data 2

Supplementary Data 3

Supplementary Data 4

## Data Availability

The data in this paper are available from the corresponding author upon reasonable request. The source data for Fig. 2b, Fig. 3a, Fig. 4a, and Fig. 4b is provided as Supplementary Data 1, Supplementary Data 2, Supplementary Data 3, and Supplementary Data 4, respectively.
