# Supplementary material for: A combined fibre/free-space-optical communication system for long-haul wireline/wireless transmission at millimetre-wave/sub-THz frequencies
Source: Commun Eng. 2023 May 3;2:18. doi: 10.1038/s44172-023-00068-1 (PMC10955945; doi:10.1038/s44172-023-00068-1)
Supplement: Supplementary file 1 — Description of Additional Supplementary Files [file 44172_2023_68_MOESM1_ESM.pdf]

Description of Additional Supplementary Files

File name: Supplementary Data 1

Description: Wavelength data of Fig. 2b from $\lambda 0$ to $\lambda 8$.

File name: Supplementary Data 2

Description: Data of Fig. 3a for measured BERs as a function of optical power transmitted to PD/UTC-PD.

File name: Supplementary Data 3

Description: Data of Fig. 4a for measured EVMs as a function of optical power transmitted to PD/UTC-PD.

File name: Supplementary Data 4

Description: Data of Fig. 4b for measured EVMs as a function of SNR.
